# Sex differences and risk factors in recurrent ischemic stroke

**DOI:** 10.3389/fneur.2023.1028431

**Published:** 2023-01-26

**Authors:** Ji Yeon Chung, Bit Na Lee, Young Seo Kim, Byoung-Soo Shin, Hyun Goo Kang

**Affiliations:** ^1^Department of Neurology, Chosun University School of Medicine, Gwangju, Republic of Korea; ^2^Department of Neurology, Research Institute of Clinical Medicine of Jeonbuk National University–Biomedical Research Institute of Jeonbuk National University Hospital, Jeonju, Republic of Korea; ^3^Department of Neurology, Wonkwang University School of Medicine, Iksan, Republic of Korea

**Keywords:** ischemic stroke, recurrent infarction, sex difference, smoking, secondary prevention

## Abstract

**Introduction:**

Recurrent ischemic stroke (RIS) is associated with increased mortality and poor outcomes. Therefore, secondary prevention is critical for reducing the risk of recurrent stroke. Previous studies have found sex differences in risk factors in patients with first-ever stroke; however, the results have been inconsistent for recurrent stroke. Therefore, this study aimed to investigate whether there are significant sex differences in the clinical characteristics and risk factors for recurrent ischemic stroke.

**Methods:**

We retrospectively studied 787 patients with recurrent ischemic stroke after first-ever stroke confirmation using magnetic resonance imaging (MRI) after visiting a regional tertiary hospital between 2014 and 2020. Demographic characteristics, laboratory findings, and risk factors were compared between the male and female patients. In addition, multivariate logistic regression was performed to identify the independent factors associated with stroke recurrence in male patients.

**Results:**

Among the 787 patients, 466 (59.2%) were males. Males were younger than females (67.6 vs. 71.9 years). Females had higher rates of hypertension, diabetes mellitus, dyslipidemia, and overweight than those of males. However, the alcohol drinking and smoking rate were significantly higher in males than that in females. There were no statistically significant sex-based differences in the laboratory findings. Among males, hypertension, alcohol drinking, smoking and dyslipidemia was a significant risk factor for ischemic stroke recurrence.

**Conclusion:**

Hypertension and dyslipidemia were significant risk factors of recurrent ischemic stroke in both genders. Smoking and alcohol drinking were significant risk factors associated with ischemic stroke recurrence in males. Therefore, smoking cessation and alcohol abstinence are recommended after the first stroke to prevent recurrent ischemic stroke especially for males. Diabetes was a significant risk factor of ischemic stroke recurrence in females. More extensive studies are needed to understand the causal relationship of each factors with ischemic stroke recurrence according to sex differences and specification of preventive management is needed.

## 1. Introduction

Stroke is a fatal condition with a high mortality rate, and an appropriate and prompt response to acute stroke is crucial (1). To lower the mortality rate, preventing primary or secondary stroke is essential to identifying and controlling the risk factors for ischemic stroke in advance (2). Known risk factors for stroke include older age (a demographic factor), diabetes, hypertension, dyslipidemia, smoking, transient ischemic attack (TIA), and heart disease (e.g., coronary artery disease and atrial fibrillation). Moreover, stroke tends to recur, and the prognosis worsens when it recurs (2). Although previous studies have presented different results, studies on the general population have shown that the probability of recurrence within 1 year was between 3 and 22%, which increased to 10–53% within 5 years (3–5).

Modifying the risk factors associated with ischemic stroke recurrence is critical in preventing recurrence. Risk factors for stroke recurrence can be classified into non-modifiable and modifiable. Age, sex, and race are non-modifiable risk factors for stroke, while hypertension, diabetes, dyslipidemia, smoking, diet therapy, and lack of physical activity are modifiable risk factors. Although many studies on modifiable risk factors have been continuously reported, there are few reports on non-modifiable risk factors, especially sex, and the results obtained so far have been inconsistent (6–8).

Multiple studies have reported differences in the initial onset of ischemic stroke between males and females. Although males generally have a higher incidence rate than that of females, some risk factors are known to be associated with the incidence of stroke in femals (9). This reportedly has been due to differences in the immune system, genetic background, endocrine system, social factors, and oral contraceptive use (10, 11); females are known to have a higher incidence of disability and dysfunction. Due to biological characteristics and female-specific risk factors, female have a higher risk of stroke, a higher chance of experiencing recurrence, and higher severity of stroke symptoms than that of males (12–14). Males and females showed differences in various risk factors for ischemic stroke, including age, carotid artery disease, hormonal changes, and cardiac arrhythmias (12). However, there are few studies on the differences in risk factors for recurrent ischemic stroke between males and females (13, 14). Based on recent reports, no significant differences in the recurrence rate of ischemic stroke and recurrence-related risk factors between males and females were found (13, 14).

Therefore, in this study, we retrospectively investigated the recurrence rate of ischemic stroke and the risk factors for recurrence. The purpose of this study was to investigate the risk factors for recurrence of ischemic stroke in each gender, and to find out whether these risk factors differed between males and females. This study targeted patients diagnosed with ischemic stroke who regularly received outpatient treatment and took appropriate medications according to doctors' instructions.

## 2. Materials and methods

### 2.1. Study subjects

This study included patients diagnosed with ischemic stroke for the first time and more than second event among patients with acute ischemic stroke (within 7 days after the onset of ischemic stroke) confirmed using magnetic resonance imaging (MRI) after visiting a regional tertiary hospital between January 2014 and December 2020. Patients with insufficient data, those not taking antiplatelets, and undocumented stroke diagnoses were excluded. The final subjects in this study were patients with recurrent ischemic stroke who visited the outpatient department regularly and could be followed up among the patients who were hospitalized due to ischemic stroke that occurred two or more times. Patients with initial (or first) onset of ischemic stroke and recurrent ischemic stroke who visited the hospital around the same time and around same ages were compared and analyzed separately in males and females.

### 2.2. Methods

This study examined risk factors by retrospectively reviewing the medical records of 797 patients with first onset ischemic stroke and 787 patients with recurrent ischemic stroke. Ischemic stroke was diagnosed by a neurologist when there was a neurological abnormality confirmed using MRI. This study excluded cases with other causes (e.g., tumor, epilepsy, and toxicity) and TIA. The subtype classification of ischemic stroke was based on the TOAST classification criteria (15). Recurrent ischemic stroke was defined as patients who developed a new neurological abnormality that persisted for 24 h after 21 days from the onset of the first ischemic stroke, patients whose existing symptoms worsened, or patients who developed a lesion at a location different from the location of the original lesion (if it occurred within 21 days among patients who were initially diagnosed with ischemic stroke and received treatment such as taking medications through regular outpatient treatment). Even if a new lesion was detected using computed tomography (CT) or MRI, asymptomatic patients were excluded (4).

This study investigated age, sex, height, weight, body mass index (BMI), abdominal circumference, peripheral arterial disease, hypertension, diabetes, dyslipidemia, coronary artery disease (CAD), TIA, smoking, and overweight as risk factors for cerebrovascular disease. Each item is defined as follows. Hypertension was defined in patients who had already been diagnosed with hypertension before hospitalization or had a systolic blood pressure of 140 mmHg or higher and a diastolic blood pressure of 90 mmHg or higher after the acute phase of ischemic stroke had passed or had already been diagnosed. If white-coat hypertension was suspected, patients were recommended to maintain a blood pressure diary at home. Diabetes was defined in patients who were diagnosed with diabetes before hospitalization, those who had fasting blood glucose of 126 mg/dL or higher (8 h after admission), those who had a blood glucose level of 200 mg/dL or higher on the random blood glucose test along with diabetes symptoms, and those who had a blood glucose level of 200 mg/dL or higher. Dyslipidemia was defined as a total cholesterol level ≥200 mg/dL or a low-density lipoprotein (LDL)-cholesterol level ≥130 mg/dL in a fasting blood test. The results of the blood test performed on the first day of hospitalization were investigated. CAD was defined as a case diagnosed by a cardiologist before or after admission or a history of percutaneous coronary intervention or bypass surgery. TIA was investigated by recording the medical history of patients or guardians using the medical records only for paroxysmal local brain dysfunctions that were caused by cerebral blood flow disorders that completely recovered within 24 h. Smokers were defined as those who smoked five cigarettes per day regularly. Persistent smokers who continued to smoke at the time of ischemic stroke recurrence after the initial onset of ischemic stroke were classified as smokers. Those who quit or had stopped smoking for more than 1 year were considered non-smokers. Alcohol consumption was classified as a risk factor when the daily alcohol intake was ≥20 mg for 3 months or longer. Overweight was defined as BMI ≥25.

This study was approved by the ethics review committee of our center. Informed consent was waived due to the retrospective nature of the study (CUH 2022-03-015). All the procedures were performed in accordance with the ethical standards of the institutional and national research committees and the Declaration of Helsinki.

### 2.3. Statistical analysis

First, demographics and laboratory findings were compared between female and male patients with first and recurrent ischemic stroke. Pearson's chi-square or Fisher's exact test was used for categorical variables, and the *t*-test was used for continuous variables. Second, multivariate analysis was performed to identify independent factors associated with stroke recurrence in each gender. To avoid variable selection caused by spurious correlations, only variables showing a potential association (*p* < 0.1) in the univariate analysis were included as potential factors associated with stroke recurrence in male patients in the multivariate logistic regression model. Statistical significance was set as *p* < 0.05 (two-tailed). All statistical analyses were performed using SPSS 21.0 (IBM Corporation, Armonk, NY, USA).

## 3. Results

### 3.1. Age and sex distribution

**Figure 1:**
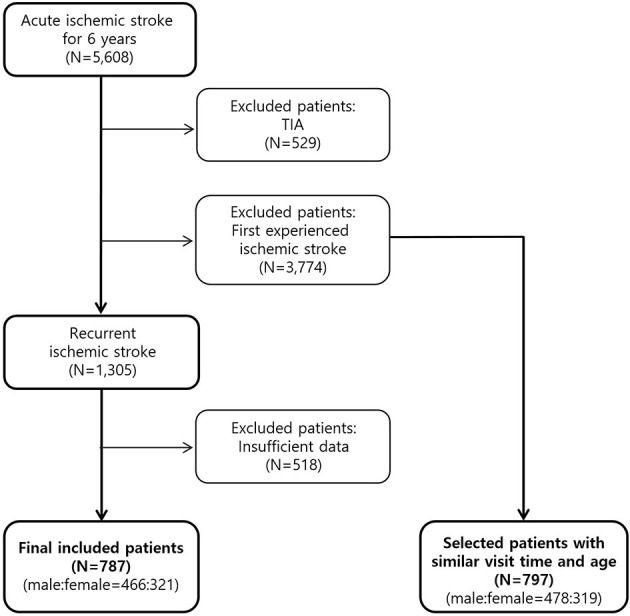
Flow diagram of recurrent ischemic stroke patient selection. First onset patients were set as a control group and both groups were compared divided by male and female.

During the study period, 5,608 patients with acute ischemic stroke were hospitalized and treated. This study excluded 3,774 patients who experienced ischemic stroke for the first time and 529 patients diagnosed with TIA. This study also excluded 518 patients with insufficient data were excluded. Finally, this study evaluated the data obtained from 787 patients (Figure 1). Among the excluded 3,774 first onset ischemic stroke, 797 patients were selected with a similar visit time and age to the 787 recurrent patients and set as a control group.

### 3.2. Differences in risk factors between male and female patients with recurrent ischemic stroke

Among the 787 patients, 466 (59.2%) were males. The mean age of the males was 67.6 years, which was lower than that of the females (71.9 years) (Table 1). Regarding the TOAST classification of patients with recurrence, the proportion of large artery atherosclerosis (LAA) was 49.6% in males and 42.1% in females, showing that the proportion was significantly higher in males (p=0.038). Females had significantly more cases of hypertension (83.3 vs. 91.0%, *p* = 0.002), diabetes mellitus (45.7 vs. 55.8%, *p* = 0.006), dyslipidemia (65.7 vs. 73.8%, *p* = 0.016), and overweight (BMI >25: 34.8 vs. 42.4%, *p* = 0.006) than those of males. In contrast, 71.1 and 7% of males and females had a smoking history, respectively, indicating that the smoking rate was significantly higher among males (*p* < 0.001).

**Table 1:** Comparison of baseline characteristics between males and females patients with recurrent ischemic stroke.

	**Males** **(*n* = 466)**	**Females (*n* = 321)**	***P-value***
Age	67.55 ± 10.51	71.87 ± 8.80	<0.001
Height (m)	166.79 ± 5.55	154.43 ± 5.44	<0.001
Weight (kg)	67.40 ± 9.87	58.52 ± 9.67	<0.001
BMI (kg/m^2^)	24.19 ± 3.04	24.55 ± 3.66	0.148
Abdominal circumference	88.79 ± 10.02	88.62 ± 11.27	0.948
**TOAST**			
LAA	231 (49.6)	135 (42.1)	0.038
SVO	235 (50.4)	186 (57.9)	
ICAS	304 (65.2)	199 (62.0)	0.352
NIHSS (admission)	4.67 ± 3.79	4.88 ± 4.05	0.465
NIHSS (discharge)	3.89 ± 4.24	4.18 ± 4.50	0.354
mRS (discharge)	2.09 ± 1.33	2.34 ± 1.29	0.087
Previous TIA or Stroke Hx	234 (50.4)	146 (45.5)	0.173
Peripheral artery disease	5 (1.1)	1 (0.3)	0.41
Coronary heart disease	66 (29.5)	32 (24.4)	0.306
HTN	388 (83.3)	292 (91.0)	0.002
DM	213 (45.7)	179 (55.8)	0.006
Dyslipidemia	305 (65.7)	237 (73.8)	0.016
Alcohol drinking	310 (66.5)	23 (7.2)	<0.001
Smoking	330 (71.1)	24 (7.6)	<0.001
Overweight (BMI > 25)	162 (34.8)	136 (42.4)	0.031
Thrombolysis	31 (6.7)	10 (3.1)	0.028
WBC (10^3^/μ*l*)	8.38 ± 3.15	7.97 ± 2.92	0.067
Hb (g/dL)	14.10 ± 1.73	12.89 ± 1.63	<0.001
Platelet (10^3^/μ*l*)	237.03 ± 71.50	264.59 ± 75.68	<0.001
BUN (mg/dL)	18.42 ± 8.44	17.61 ± 7.33	0.168
Creatinine (mg/dL)	1.01 ± 0.59	0.80 ± 0.76	<0.001
Total cholesterol (mg/dL)	162.74 ± 39.73	177.39 ± 47.37	<0.001
Triglyceride (mg/dL)	133.28 ± 79.33	126.20 ± 83.78	0.237
HDL-cholesterol (mg/dL)	40.44 ± 17.66	45.11 ± 11.62	<0.001
LDL-cholesterol (mg/dL)	102.93 ± 37.94	113.21 ± 42.09	0.001
HbA1c (%)	6.66 ± 1.46	6.76 ± 1.54	0.348
CRP (mg/dL)	1.42 ± 8.73	1.27 ± 2.73	0.763
Fibrinogen	320.25 ± 83.49	342.61 ± 98.45	0.001

Results expressed as number (% column) or mean (standard deviation).

BMI, body mass index; LAA, large artery atherosclerosis; SVO, small vessel occlusion; ICAS, intracranial arterial stenosis; NIHSS, National Institutes of Health Stroke Scale; mRS, modified Rankin scale; TIA, transient ischemic attack; HTN, hypertension; DM, diabetes mellitus. WBC, white blood cell; Hb, hemoglobin; BUN, blood urea nitrogen; HDL, high-density lipoprotein; LDL, low-density lipoprotein; HbA1c, hemoglobin A1c; CRP, C-reactive protein.

### 3.3. Differences in laboratory findings between male and female patients with recurrent ischemic stroke

Hemoglobin, platelet, creatinine, total cholesterol, high-density lipoprotein (HDL), LDL, fibrinogen, and clopidogrel resistance levels were significantly different between males and females (Table 1). In addition, hemoglobin, creatinine, and clopidogrel resistance rates were significantly higher in males, while PLT, TC, HDL, LDL, and fibrinogen levels were significantly higher in females (Table 1). However, other laboratory findings were not significantly different between males and females.

### 3.4. Differences in risk factors and laboratory findings between first and recurrent ischemic stroke in males

ICAS was significantly higher in first stroke, and coronary heart disease, HTN, DM, dyslipidemia, alcohol drinking, smoking were significantly higher in recurrent ischemic stroke. Hemoglobin, platelet, total cholesterol were significantly higher in recurrent ischemic stroke in males (Table 2).

**Table 2:** Comparison of baseline characteristics between first ischemic stroke and recurrent ischemic stroke patients in male patients.

	**First stroke (*n* = 478)**	**Recurrent stroke** **(*n* = 466)**	***P-value***
Age	68.47 ± 12.03	67.55 ± 10.51	0.213
BMI (kg/m^2^)	24.18 ± 3.18	24.19 ± 3.04	0.943
**TOAST**			
LAA	183 (61.8)	231 (49.6)	0.001
SVO	113 (38.2)	235 (50.4)	
ICAS	204 (80.6)	304 (65.2)	<0.001
NIHSS (admission)	3.87 ± 3.98	4.67 ± 3.79	0.002
NIHSS (discharge)	3.13 ± 5.70	3.89 ± 4.24	0.025
mRS (discharge)	2.36 ± 1.49	2.09 ± 1.33	0.009
Coronary heart disease	37 (7.7)	66 (29.5)	<0.001
HTN	247 (51.8)	388 (83.3)	<0.001
DM	155 (32.5)	213 (45.7)	<0.001
Dyslipidemia	106 (22.2)	305 (65.7)	<0.001
Alcohol drinking	168 (35.4)	310 (66.5)	<0.001
Smoking	145 (30.6)	330 (71.1)	<0.001
Overweight (BMI > 25)	174 (36.6)	162 (34.8)	0.566
Thrombolysis	84 (17.6)	31 (6.7)	<0.001
Hb (g/dL)	13.53 ± 2.15	14.10 ± 1.73	<0.001
Platelet (10^3^/μ*l*)	220.57 ± 72.82	237.03 ± 71.50	<0.001
BUN (mg/dL)	18.32 ± 10.00	18.42 ± 8.44	0.869
Creatinine (mg/dL)	1.06 ± 0.77	1.01 ± 0.59	0.274
Total cholesterol (mg/dL)	175.11 ± 48.12	162.74 ± 39.73	<0.001
Triglyceride (mg/dL)	155.78 ± 129.91	133.28 ± 79.33	0.002
HDL-cholesterol (mg/dL)	43.55 ± 12.31	40.44 ± 17.66	0.002
LDL-cholesterol (mg/dL)	106.09 ± 39.85	102.93 ± 37.94	0.219
HbA1c (%)	7.44 ± 23.33	6.66 ± 1.46	0.475
Fibrinogen	323.40 ± 83.57	320.25 ± 83.49	0.571

BMI, body mass index; TOAST, trial of org 10,172 in aucte stroke treatment; LAA, large artery atherosclerosis; SVO, small vessel occlusion; ICAS, intracranial arterial stenosis; NIHSS, National Institutes of Health Stroke Scale; mRS, modified Rankin scale; TIA, transient ischemic attack; HTN, hypertension; DM, diabetes mellitus. WBC, white blood cell; Hb, hemoglobin; BUN, blood urea nitrogen; HDL, high-density lipoprotein; LDL, low-density lipoprotein; HbA1c, hemoglobin A1c; CRP, C-reactive protein.

### 3.5. Differences in risk factors and laboratory findings between first and recurrent ischemic stroke in females

BMI, ICAS, coronary heart disease, HTN, DM, dyslipidemia were significantly higher in recurrent ischemic stroke. Hemoglobin, platelet, HbA1c were significantly higher in recurrent ischemic stroke in females (Table 3).

**Table 3:** Comparison of baseline characteristics between first ischemic stroke and recurrent ischemic stroke patients in female patients.

	**First stroke (*n* = 319)**	**Recurrent stroke (*n* = 321)**	***P-value***
Age	73.52 ± 12.62	71.87 ± 8.80	0.056
BMI (kg/m^2^)	23.38 ± 3.72	24.55 ± 3.66	<0.001
**TOAST**			
LAA	103 (55.7)	135 (42.1)	0.003
SVO	82 (44.3)	186 (57.9)	
ICAS	158 (94.6)	199 (62.0)	<0.001
NIHSS (admission)	5.10 ± 4.70	4.88 ± 4.05	0.538
NIHSS (discharge)	4.83 ± 7.45	4.18 ± 4.50	0.213
mRS (discharge)	2.77 ± 1.62	2.34 ± 1.30	0.001
Coronary heart disease	24 (7.5)	32 (24.4)	<0.001
HTN	200 (62.9)	292 (91.0)	<0.001
DM	92 (28.9)	179 (55.8)	<0.001
Dyslipidemia	68 (21.4)	237 (73.8)	<0.001
Alcohol drinking	27 (8.5)	23 (7.2)	0.518
Smoking	7 (2.2)	24 (7.6)	0.002
Overweight (BMI > 25)	1 (20.0)	136 (42.4)	0.403
Thrombolysis	63 (19.9)	10 (3.1)	<0.001
Hb (g/dL)	12.09 ± 1.69	12.89 ± 1.63	<0.001
Platelet (10^3^/μ*l*)	233.71 ± 79.12	264.59 ± 75.68	<0.001
BUN (mg/dL)	17.65 ± 9.37	17.61 ± 7.33	0.960
Creatinine (mg/dL)	0.80 ± 0.51	0.80 ± 0.76	0.982
Total cholesterol (mg/dL)	174.99 ± 49.12	177.39 ± 47.37	0.537
Triglyceride (mg/dL)	133.14 ± 104.98	126.20 ± 83.78	0.368
HDL-cholesterol (mg/dL)	47.82 ± 13.07	45.11 ± 11.62	0.007
LDL-cholesterol (mg/dL)	106.89 ± 40.85	113.21 ± 42.09	0.060
HbA1c (%)	6.22 ± 1.26	6.76 ± 1.54	<0.001
Fibrinogen	324.34 ± 80.08	342.61 ± 98.45	0.012

BMI, body mass index; TOAST, trial of org 10,172 in aucte stroke treatment; LAA, large artery atherosclerosis; SVO, small vessel occlusion; ICAS, intracranial arterial stenosis; NIHSS, National Institutes of Health Stroke Scale; mRS, modified Rankin scale; TIA, transient ischemic attack; HTN, hypertension; DM, diabetes mellitus. WBC, white blood cell; Hb, hemoglobin; BUN, blood urea nitrogen; HDL, high-density lipoprotein; LDL, low-density lipoprotein; HbA1c, hemoglobin A1c; CRP, C-reactive protein.

### 3.6. Risk factors for ischemic stroke recurrence in males

This study identified significant (*p* < 0.1) variables in the univariate analysis and confirmed factors associated with ischemic stroke recurrence in males using multivariate analysis to identify factors associated with the ischemic stroke recurrence by sex. The results showed that HTN, alcohol drinking, smoking, dyslipidemia were risk factors for ischemic stroke recurrence in males (Table 4).

**Table 4:** Factors related to recurrent ischemic stroke in males and females.

	**Univariate analysis**		**Multivariate analysis**	
	**Crude OR (95% CI)**	***P-value***	**Adjust OR (95% CI)**	***P-value***
**Male patients**				
HTN	4.63 (3.42–6.27)	<0.001	4.86 (3.34–7.08)	<0.001
DM	1.75 (1.34–2.28)	<0.001	1.11 (0.79–1.56)	0.552
Alcohol drinking	3.62 (2.77–4.74)	<0.001	2.41 (1.73–3.34)	<0.001
Smoking	5.59 (4.22–7.39)	<0.001	5.11 (3.65–7.16)	<0.001
Dyslipidemia	6.71 (5.03–8.96)	<0.001	5.03 (3.61–7.02)	<0.001
**Female patients**				
BMI	1.09 (1.04–1.14)	<0.001	1.02 (0.96–1.07)	0.553
HTN	5.94 (3.81–9.26)	<0.001	3.71 (2.20–6.25)	<0.001
DM	3.09 (2.23–4.30)	<0.001	2.01 (1.23–3.28)	0.006
Dyslipidemia	10.37 (7.19–14.95)	<0.001	8.15 (5.49–12.09)	<0.001
HbA1c	1.34 (1.18–1.52)	<0.001	1.03 (0.87–1.23)	0.726
Smoking	3.62 (1.54–8.52)	0.003	2.76 (0.98–7.80)	0.055

Non-parametric tests were performed for continuous variables that did not show normal distribution and are presented as median (25–75 percentile range).

Results are expressed as odds ratios (ORs) and 95% confidence intervals (CIs). Variables with p < 0.1 by univariate analysis were entered into the multivariate analysis model. HTN, hypertension; DM, diabetes mellitus; HbA1c, hemoglobin A1c, LDL, low-density lipoprotein; BMI, body mass index.

### 3.7. Risk factors for ischemic stroke recurrence in females

This study identified significant (*p* < 0.1) variables in the univariate analysis and confirmed factors associated with ischemic stroke recurrence in females using multivariate analysis to identify factors associated with the ischemic stroke recurrence by sex. The results showed that HTN, DM, dyslipidemia were risk factors for recurrence of ischemic stroke in females (Table 4).

## 4. Discussion

Undoubtedly, recurrent stroke is a dangerous and frightening event in stroke patients. In particular, as medical sciences develop, the proportion of the elderly population and mean age have rapidly increased. Simultaneously, much attention has been given to improving the quality of life of the elderly population. Many studies have evaluated primary and secondary prevention programs for stroke in various countries owing to the surge in socioeconomic costs for stroke patients. Many studies on stroke recurrence have reported different results, depending on the methods, subjects, and analyses (6–8).

Although there have been many studies on the recurrence rate, risk factors, and incidence rates between males and females with ischemic stroke, they have shown different inconsistent results (6–8, 16). Most studies have reported no significant difference in recurrence factors between males and females (6–8, 16). However, Jung et al. (16) revealed that males had a higher overall recurrence rate and females had a higher chance of earlier recurrence. The males-to-females ratio was 1.45:1. Since the results between the two groups were not statistically significant, it was impossible to conclude that males were more likely to experience recurrence than females in this study. Basu et al. (13) reported in 2021 that risk factors associated with recurrence did not differ between males and females with ischemic stroke and transient ischemic stroke.

Although hypertension has been established as one of the causative factors of stroke, various views regarding its effect on recurrence have been discussed. Sacco et al. (2), Lai et al. (3), Jorgensen et al. (5), and Hier et al. (17) reported that hypertension was significantly associated with recurrence, whereas Petty et al. (18) reported no significant relationship. This study showed significant association between higher risk of recurrence with a history of hypertension and hypertension diagnosed during hospitalization in both genders, which is similar to previous findings (2, 3, 5, 17).

Diabetes is a strong risk factor for stroke, with evidence from previous meta-analyses to suggest that the risk of stroke associated with diabetes is greater in women than men, independently of other stroke risk factors (19), but the relationship between diabetes and stroke recurrence is controversial. Although many studies reported that the incidence rate of stroke is high in diabetic patients, Alter et al. (20) revealed that the follow-up of diabetes patients and recurrence rates for 2 years were not significantly different compared to those of the non-diabetic group. Alter et al. (20) also showed that in a 4-year follow-up, diabetes did not affect the recurrence rate of ischemic stroke despite blood glucose dysregulation. In this study, although not statistically significant in the male group, diabetes was identified as a risk factor for ischemic stroke recurrence in females (Table 1).

Dyslipidemia is an important risk factor for ischemic stroke. In this study, dyslipidemia was significantly associated with higher risk of recurrent ischemic stroke in both genders. 65% of males and 73% of females with recurrent ischemic stroke had a history of dyslipidemia, which showed that the proportion was higher in females than that in males. Furthermore, total cholesterol and LDL cholesterol levels were also significantly higher in females than those in males, consistent with that reported by Chen et al. (21), who showed that femalse with recurrent ischemic stroke had significantly higher cholesterol levels than those in males with recurrent ischemic stroke. This is attributed to an increase in LDL cholesterol, associated with a decrease in estrogen levels in postmenopausal females (22).

Although smoking is recognized as a risk factor for ischemic stroke, there is insufficient evidence suggesting that it is a causal factor of recurrence. In this study, the smoking rate among patients with recurrent ischemic stroke was significantly higher in males than that in females (*p* < 0.001, Table 1). Since smoking among females continues to be perceived negatively in many countries in East Asia due to social norms, smoking among males may be very high. However, smoking affects the recurrence of ischemic stroke in males significantly. Several studies have reported smoking as a risk factor for stroke. Smoking after the onset of the initial stroke would increase the risk of stroke recurrence, and a dose-response relationship was ascertained with the amount of smoking (21). However, Boiten and Lodder (23) and the World Health Organization (24) reported that smoking was not significantly associated with recurrence. Owing to these contrary results, the effect of smoking on the recurrence of ischemic stroke remains unclear and difficult to explain. However, the results of this study confirmed smoking as an important risk factor for recurrent ischemic stroke. Since this study only investigated whether patients smoked within 1 year from the onset of stroke without considering the duration and amount of smoking to identify smoking history, further detailed research is needed.

Alcohol is known to reduce stroke risk when consumed in moderation, but increases risk of ischemic stroke when consumed in excess. In particular, it is known that there is a close relationship between the occurrence of hemorrhagic stroke and alcohol (2). However, the association with ischemic stroke recurrence is not well-known. In this study, similar with the smoking rate, the rate of alcohol intake among patients was higher in males significantly and was associated with higher risk of ischemic stroke recurrence in males, but not in females.

This study had several limitations. First, since the study was conducted retrospectively on a group of patients who regularly visited a university hospital rather than a community-based group, there was a possibility of selection bias because the risk factors for ischemic stroke could be controlled to some extent for these patients. Furthermore, because this study was based on outpatients, patients had different follow-up intervals and different numbers and intervals of examinations. Consequently, evaluating the effects of treatment methods and efficacy was difficult. Although this study identified that smoking was one of risk factors for recurrent ischemic stroke in males, we did not know the type of cigarette, age since smoking, attempts to quit, duration of cessation, duration of smoking, amount of cigarette consumption, and depth of inhalation. Future studies should examine the dose-response relationship between smoking quantity and stroke recurrence in the group that continued to smoke after the onset of the initial ischemic stroke. We also believe that comparing differences between a group that continued to smoke after the first stroke and one that stopped smoking after the incident and comparing the smoking rate between first stroke and recurrent stroke would yield more solid evidence for recommending smoking cessation for the secondary prevention of ischemic stroke. Since the rate of smoking among young females is on the rise in recent years, if the smoking rate between males and females becomes similar in the future, it is highly likely that similar results will be obtained not only for males but also for females.

Since the results of this study revealed that smoking and alcohol drinking is distinct risk factors for the recurrence of ischemic stroke in males, it is believed that smoking cessation and alcohol abstinence will be especially beneficial for the secondary prevention of ischemic stroke in males who have experienced an onset of ischemic stroke. Although the American Heart Association announced in 2014 that smoking cessation was a class I recommendation in secondary prevention guidelines for stroke, the level of evidence was only C (25). There is yet not enough evidence to support this recommendation. Therefore, more extensive studies are needed to understand the effect of smoking cessation on secondarily preventing ischemic stroke in males.

In this study, it was found that hypertension and dyslipidemia are significant risk factors for recurrence of ischemic stroke in both genders. Factors differed between genders were smoking and drinking in males and diabetes in females. Therefore, as a secondary prevention of ischemic stroke, hypertension and dyslipidemia should be controlled importantly, and factors differed between genders should be intensively controlled according to sex differences.

## Data availability statement

The original contributions presented in the study are included in the article/supplementary material, further inquiries can be directed to the corresponding author.

## Ethics statement

This study protocol was reviewed and approved by the Institutional Review Board of Chosun University Hospital (approval number: CUH 2022-03-015). Written informed consent for participation was not required for this study in accordance with the national legislation and the institutional requirements.

## Author contributions

JC and BL contributed to the study concept and design, data collection and interpretation, manuscript drafting, and revision. YK and B-SS contributed to the data interpretation and revised the manuscript. HK contributed to the study concept and design, data interpretation, manuscript drafting, and revision. All authors contributed to the article and approved the submitted version.
